# Free DICOM browsers

**DOI:** 10.4103/0971-3026.38503

**Published:** 2008-02

**Authors:** Dandu Ravi Varma

**Affiliations:** Department of Radiology, Krishna Institute of Medical Sciences, 1-8-31/1, Minister Road, Secunderabad - 500 003, Andhra Pradesh, India

## DICOM and DICOM Browsers

The digital imaging and communications in medicine (DICOM) standard was created by the National Electrical Manufacturers' Association (NEMA) in order to improve compatibility and workflow efficiency between imaging systems, medical devices, and other information systems used in a hospital environment. This has become the principal standard for the communication of medical images and is now implemented by virtually all medical imaging equipment manufacturers.

Image files that conform to the DICOM standards are commonly referred to as DICOM-format images. The basic difference between a DICOM image and an image in other formats like JPEG, TIFF, or GIF is that the DICOM image contains a ‘header’ with information (such as patient demographics, machine, scan parameters, and a host of other non-image data) in addition to the image data. Thus it is possible to identify the origin of the image, the patient, data acquisition parameters of the study and so on, even if a single image is analyzed in isolation. The adoption of DICOM standards by medical imaging equipment vendors has helped in effective cross-machine communications and made possible integration of imaging equipment from different manufacturers.[1]

Although DICOM is the accepted standard for the storage and networking of medical images, the image is encoded in a format that cannot readily be viewed on an average personal computer (PC). Viewing these images outside the radiology network still requires printing of the images on hardcopy films. With the fall in prices of hardware and peripheral devices, computers with high-end hardware specifications are available at affordable prices. To view DICOM images on PCs, dedicated DICOM browsers are needed. Though these softwares are available from several vendors, they are expensive and beyond the reach of the average radiologist.

Several freely downloadable DICOM browsers, which can be easily integrated into a radiologist's practice, are available via the Internet.[2–4] This article describes the applications of a few DICOM browsers in clinical radiology and imaging research and evaluates their merits and demerits in common radiology applications (Appendix 1).

## Free DICOM Browsers Available Online: A Functional Classification

The DICOM browsers can be broadly and functionally segregated into four groups: for viewing DICOM images, for teaching, for use as mini-PACS servers, and for research purposes. Let us examine each of these individually.

### DICOM browsers for basic viewing of DICOM images

The simplest and most intuitive application of DICOM browsers is to view images on stand-alone systems that are not a part of a radiology network. Most equipment manufacturers offer the option of archiving imaging data on compact discs (CDs) which can be transported, stored, and reviewed easily. Though a proprietary DICOM viewer is written into the CD along with the image data, it is often limited in its functionality. Using a DICOM browser of one's own choice permits the use of a set of tools suited to one's needs, irrespective of the source of the imaging data. It must be remembered that most freely downloadable software is intended for educational purposes and is not custom built for medical diagnosis or commercial purposes.

Most browsers in this category [Table 1] provide basic image-processing functions, such as the ability to adjust image contrast and brightness, to magnify, to perform measurements (distances, angles, areas, pixel values, etc.), to change image orientation, and the ability to annotate. Studies containing a time series of image data need a ‘cine’ viewer with the ability to change the frame rate. Another useful tool is the ability to simultaneously load and compare two sets of images (available with Jivex [dv] viewer and Sante DICOM viewer); this is usually necessary when looking for enhancement on post-contrast studies and to check for response to therapy. The ability to view the localizer/scout view, along with a specific image, is a feature of Sante DICOM and Jivex [dv] viewers as well.

**Table 1:** Free DICOM browsers - basic viewing software

Free DICOM software	Web address	Free/limits	DICOM query	Thumbnail view	Display layout	Flip/rotation	Invert b/w	Linear measure	Angle measure	Cine viewer	Compare view	Scout localizer support	Software required	Additional features
3D-Doctor Viewer v 4.0.070803	http://www.ablesw.com/3d-doctor/get3dview.html	Free	No	No	1 × 1	No	No	No	No	Yes	No	No	None	Full version is an advanced 3D visualization and processing tool
CarDiCon v 1.53B	http://fhs-consulting.com/cardicon/E_cardicon.html	Free	No	Yes	1 × 1	No	No	No	No	Yes	Yes	No	None	Primarily a browser for angiographic studies
DCM View	http://www.rmrsystems.co.uk/dcmview.htm	Free	No	Yes	1 × 1 to all-in-one	No	No	No	No	No	No	No	None	No installation. Small program
DCM Vista v 3.2	http://www.charruasoft.com/downen.php	Free	Yes and data import function	Yes	1 × 1 to 4 × 5	Yes	Yes	Yes	No	Yes	No	No	None	No installation. Small program
Dicomlight v 5.4	http://www.charruasoft.com/downen.php	Free	No	No	1 × 1 to 4 × 5	No	Yes	No	No	No	No	No	None	No installation. Small program
DICOM Scope v 3.5.1	http://dicom.offis.de/dscope.php.en	Free	No	No	1 × 1	Yes	Yes	Yes	Yes	No	No	No	Java	Offers structured reporting
ImRead DICOM Viewer	http://www.uchsc.edu/sm/neuroimaging/download/imread/imread1.htm	Free	No	No	1 × 1	Yes	No	No	No	No	No	No		
Tomovision	http://www.tomovision.com/	Can open only five images at a time	No	No	1 × 1	No	No	No	No	Yes	No	No		

### DICOM browsers useful for teaching

Of late, more and more departments are beginning to use electronic media for teaching. The rising cost of x-ray films and the ease with which digital images can be archived, queried, retrieved, and presented, have seen the gradual replacement of the ‘departmental film library’ by a ‘virtual library’ of ‘teaching collections.’ The greatest advantage of creating digital teaching files is the ease with which they can be stored and distributed.

The database of a single server can hold much more image data than several rooms containing shelves and shelves of ageing and disintegrating radiographs and this data can be accessed simultaneously by several users across a network or over the World Wide Web. The addition of non-radiological medical images, such as clinical photographs, endoscopic views, and photomicrographs, enables the creation of a comprehensive multimodality teaching file.

Images in DICOM format are usually converted to other image formats such as JPEG, GIF, or TIFF prior to creation of the teaching file. These formats are more compact than the original image and are recognized by most non-medical image management software that can be used to create teaching files and albums. Software for this purpose must have the capability to adjust image quality, size, and resolution [Table 2]. Batch conversion of a series of images saves the operator from having to vary the window level and width in individual images; (this feature is available with DicomWorks.) The ability to produce movie files from a series of individual images (a feature available with CarDiCon and DicomWorks) and to visualize 3D data (which is possible with 3D Doctor Viewer and FP Image Viewer) are an added advantage.[5]

**Table 2:** Free DICOM browsers useful for teaching

Free DICOM Software	Web address	Input format	Thumbnail view	Display layout	Flip/rotate	Invert b/w	Text annotations	Measurements	Cine viewer	Export Formats	Annotations Off	Additional software required	Additional features
Radfiler DICOM for PowerPoint	http://www.radfiler.com/	DICOM	No	1 × 1	No	No	No	No	No	No	No	Microsoft PowerPoint	Provides DICOM functionality within a presentation
DicomLitev1.01	http://www.3d-head.com	DICOM	No	1 × 1	No	No	No	No	No	TIFF	No	None	No installation. Small program
DCM2JPG	http://www.sph.sc.edu/comd/rorden/ezdicom.html	DICOM	No	No	No	No	No	No	No	BMP, JPEG	No	None	Command line conversion to JPG/BMP formats
DICOM Browser from FP Image	http://www.fpimage.com/info.html	DICOM	No	1 × 1	No	No	No	No	No	JPEG	Yes		
DicomWorks v 1.3.5	http://dicom.online.fr/	DICOM	No	1 × 1	No	No	No	No	Yes	JPEG, AVI	Yes		MPR and CMPR Recon of 3D data, Direct e-mail of images
Eviewbox	http://eviewbox.sourceforge.net/	DICOM	Yes	Auto	No	No	No	No	Slideshow	GIF	No	Java	MPR and CMPR Recon of 3D data, Direct e-mail of images
ezDICOMv1.0	http://www.sph.sc.edu/comd/rorden/ezdicom.html	DICOM, Medical image formats	No	1 × 1	No	Yes	No	No	No	BMP, JPEG Medical image formats	Yes	None	Primarily aimed at converting data into other medical image formats
ImageJ v 1.38x	http://rsb.info.nih.gov/ij/	DICOM	No	1 × 1	Yes	No	Yes	Yes	Yes	JPEG, GIF, TIFF, BMP, PNG, AVI	No	Java	Image Stacking, reslicing, Z-project, 3D Project,
ImRead DICOM Processor	http://www.uchsc.edu/sm/neuroimaging/download/imread/imread1.htm	DICOM	No	1 × 1	Yes	No	No	No	No	JPEG, TIFF	No	Java	Small program
IrfanView v 4.1	http://www.irfanview.com/	DICOM	No	1 × 1	Yes	Yes	No	No	Slide-show	Can be converted into a large number of image formats	No	Additional plugin	Image management software with DICOM image support
JiveX [dv] Viewer v 4.2.1	http://www.visus-tt.com/DICOM_Viewer.59.0.html	DICOM images	Yes	1 × 1 to 4 × 4	Yes	Yes	Yes	Yes	Yes	image, matrix, series disabled	Yes	Java version 1.4	Save presentation state to unique format; needs key
MEDISP v 1.0.2	http://medisp.bme.teiath. gr/downloadMEDISPDI-COMViewer.htm	DICOM	Yes	1 × 1 to 4 × 4	Yes	Yes	Yes	Yes	Yes	JPEG, TIFF, BMP, PNG, AVI	Yes	None	
Osiris v 4.19	http://www.sim.hcuge. ch/osiris/01 _Osiris_Presentation_EN.htm	DICOM	No	1 × 1	Yes	Yes	Yes	Yes	Yes	JPEG, BMP, TIFF, AVI	Yes	Yes	No

An earlier publication has described the integration of DICOM viewer functions into Microsoft PowerPoint presentations by the installation of ActiveX components and add-ins.[6] A freely available software - Iconotech[7] - is now available to help compile a database of teaching files, incorporating patient demographic information, clinical details, imaging findings, diagnosis, as well as bibliographic references, along with DICOM images. Presentation as teaching cases as well as in the form of quizzes, is enabled in this software.

It must always be remembered that when an image is being stored for teaching purposes or for publication, it is essential to hide the demographic information on the image. In most browsers it is possible to turn annotations off while the image is being exported into a different format, to maintain patient confidentiality. Some browsers also have the ability to alter certain tags in the DICOM header so as to introduce information into the image to help in organizing the teaching file.

### DICOM browsers for mini-PACS servers

A direct extension of the capabilities of a DICOM browser would be the ability to access, store and retrieve, transmit, display, and process images that were generated by DICOM-compatible equipment. Softwares in this category range from simple solutions called mini-PACS to extremely complex systems that interact with the hospital information system/radiology information system (HIS/RIS), electronic medical record systems, and web servers. Essential features of such softwares include the ability to access DICOM data available on a local or network drive and to search for DICOM data within hard disks, folders, and CDs, using patient name, study number, study date, or other queries. Examples of software available on the web that can be used for such applications include CharuaPACS,[8] Dicomscope,[9] KPACS,[10] Tudor DICOM Viewer,[11] and Sante DICOM Viewer.[12]

The setting up of such a system and its integration with the imaging equipment usually requires the help of a network administrator. Such software can also be of use for arranging viewing stations for monitoring imaging studies from the radiologist's office, for printing images using DICOM printers or paper printers, and for teleradiology.

### DICOM Browsers for Research Applications

Anonymization of the DICOM header is of vital importance in medical research, where the identity of the patient and the participating institution needs to be removed from the DICOM header [Table 3]. There are several software programs that can be used for extraction and analysis of structural and functional imaging data. These programs have been developed for applications such as image segmentation, registration with atlases, contour generation, texture analysis, computer-aided diagnosis, volumetric studies, and relaxometric studies. As some of these programs are designed to run on image data in non-DICOM formats (such as Analyze and NIFTI), DICOM data needs to be converted using applications such as MRIcro. A review on freely downloadable software for offline analysis of structural and functional neuroimaging data can be found in a future issue of this journal.

**Table 3:** Free DICOM browsers useful for research

Free DICOM Software	Web address	Free/limits	Input format	DICOM query	View DICOM Info	Edit DICOM Info	Anonymize	Export function
DICOM Browser FP Image	http://www.fpimage.com/info.html	Free	DICOM	No	Yes	No	Yes	JPEG
DicomWorks v 1.3.5	http://dicom.online.fr/	Free	DICOM	Yes	Yes	No	Yes	JPEG, AVI
MEDISPv 1.0.2	http://medisp.bme.teiath.gr/downloadMEDISPDICOMViewer.htm	Free	DICOM	Yes	Yes	No	Yes	JPEG, TIFF, BMP, PNG, AVI
MRIcro v 1.4	http://www.mricro.com	Free	DICOM and other formats	Yes	Yes	No	Yes	Conversion into a wide variety of medical image formats for functional analysis
Same DICOM Viewer 6 (Demo mode)	http://users.forthnet.gr/ath/mkanell/	Unlimited time with watermarks and options disabled	DICOM and DICOMDIR	Yes	Yes	No	Yes	Watermarked JPEG, TIFF, BMP, PNG, HTML, AVI

## Summary

There is a large array of DICOM browsers that can be downloaded from the Net free of cost. These can prove to be effective alternatives to commercially available programs and radiologists can implement these solutions to augment their clinical practice, taking into consideration their individual requirements as well as the software's functional capabilities and limitations.

## Appendix 1: Testing Methodology of Free DICOM Browsers

1. For the preparation of this article, software for viewing and processing DICOM image data, that can be downloaded free of cost, was evaluated on a notebook computer with a 1.73 GHz Intel Centrino processor, 1GB RAM, and Microsoft Windows XP Professional operating system.
2. As on 25 Oct 2007, most browsers were available as a simple download link, while a few required online registrations to unlock certain features. All programs were simple to install. A few programs required the installation of additional software (such as Java Runtime Environment) that is available on the web.
3. Softwares that require compilation before use, handle proprietary and non-DICOM medical image formats, and run on other operating systems have been excluded from the review.
4. All softwares were evaluated independently by an experienced radiologist and a trainee radiologist for features such as ease of installation, range of tools available, and the relative ease of operation. Though some browsers were able to process non-DICOM image formats as well as proprietary image formats, these capabilities were ignored for the purpose of this review.
5. Though every attempt has been made to keep the information about these browsers accurate and up-to-date, variations in host computer specifications and periodic software updates may cause inconsistent browser functionality. Readers are urged to study the relevant up-to-date documentation regarding these browsers.
